# Identification of the central role of RNA polymerase mitochondrial for angiogenesis

**DOI:** 10.1186/s12964-024-01712-9

**Published:** 2024-06-21

**Authors:** Meng-Jia Huan, Ping-ping Fu, Xia Chen, Zhao-Xia Wang, Zhou-rui Ma, Shi-zhong Cai, Qin Jiang, Qian Wang

**Affiliations:** 1 Department of Ophthalmology, The Second Affiliated Hospital of Nanjing Medical University, Nanjing, China; 2 The Affiliated Eye Hospital, Nanjing Medical University, Nanjing, 210029 China; 3 Department of Ophthalmology, Shanghai Eye Diseases Prevention & Treatment Center, Shanghai Eye Hospital, School of Medicine, Tongji University, Shanghai, China; 4 Department of Anesthesiology, Children’s hospital of Soochow University, Suzhou, 215025 China; 5 Department of Endocrinology, Fengcheng Hospital of Fengxian Distric, Shanghai, China; 6 Department of Child and Adolescent Healthcare, Children’s Hospital of Soochow University, Suzhou, China; 7 Key Laboratory of Congenital Structural Malformations of Suzhou City, Suzhou, China; 8 Department of Burn and Plastic Surgery, Children’s Hospital of Soochow University, Suzhou, China

## Abstract

Mitochondria are central to endothelial cell activation and angiogenesis, with the RNA polymerase mitochondrial (POLRMT) serving as a key protein in regulating mitochondrial transcription and oxidative phosphorylation. In our study, we examined the impact of POLRMT on angiogenesis and found that its silencing or knockout (KO) in human umbilical vein endothelial cells (HUVECs) and other endothelial cells resulted in robust anti-angiogenic effects, impeding cell proliferation, migration, and capillary tube formation. Depletion of POLRMT led to impaired mitochondrial function, characterized by mitochondrial depolarization, oxidative stress, lipid oxidation, DNA damage, and reduced ATP production, along with significant apoptosis activation. Conversely, overexpressing POLRMT promoted angiogenic activity in the endothelial cells. In vivo experiments demonstrated that endothelial knockdown of POLRMT, by intravitreous injection of endothelial specific POLRMT shRNA adeno-associated virus, inhibited retinal angiogenesis. In addition, inhibiting POLRMT with a first-in-class inhibitor IMT1 exerted significant anti-angiogenic impact in vitro and in vivo. Significantly elevated expression of POLRMT was observed in the retinal tissues of streptozotocin-induced diabetic retinopathy (DR) mice. POLRMT endothelial knockdown inhibited pathological retinal angiogenesis and mitigated retinal ganglion cell (RGC) degeneration in DR mice. At last, POLRMT expression exhibited a substantial increase in the retinal proliferative membrane tissues of human DR patients. These findings collectively establish the indispensable role of POLRMT in angiogenesis, both in vitro and in vivo.

**Supplementary Information:**

The online version contains supplementary material available at 10.1186/s12964-024-01712-9.

## Introduction

Endothelial cells are pivotal orchestrators of angiogenesis, a tightly regulated and multifaceted biological process that entails the formation of new blood vessels from pre-existing ones [1–5]. It commences with the degradation of the basement membrane and is followed by the activation, proliferation, and migration of endothelial cells [1–5], ultimately culminating in the formation of new blood vessels and vascular networks [1–5]. Comprehensive understanding of the molecular mechanisms underpinning endothelial cell-mediated angiogenesis has yielded insights into potential therapeutic strategies for diseases marked by aberrant vascularization, including the development of anti-angiogenic interventions aimed at disrupting this process to impede disease progression [1–5].

Energy demand plays a critical role in endothelial cell activation and angiogenesis [6, 7]. Mitochondria, the powerhouses of eukaryotic cells, are responsible for oxidative phosphorylation (OXPHOS) and energy production [8, 9]. Besides energy generation, mitochondria are involved in regulating essential cellular processes, including cell differentiation, signal transduction, apoptosis, cell growth, and the cell cycle [10–15]. Maintaining functional mitochondria is crucial for preserving mitochondrial membrane potential, membrane channel integrity, and Ca^2+^ concentration [7, 16], all of which are pivotal for endothelial cell activation and angiogenesis [7, 16]. Conversely, mitochondrial dysfunction leads to mitochondrial depolarization, reactive oxygen species (ROS) production, oxidative stress, Ca^2+^ overload, ATP depletion, energy crisis, and cell apoptosis [7, 16–19], all of which hinder endothelial cell activation and block angiogenesis [7, 16–19]. Therefore, endothelial mitochondria play a central role in promoting angiogenesis.

In our previous research, we have been dedicated to identifying key mitochondrial proteins essential for angiogenesis. Recently, we demonstrated the significance of TIMM44 (translocase of inner mitochondrial membrane 44), a pivotal protein for mitochondrial functions and integrity, in angiogenesis [20]. Silencing TIMM44 using targeted shRNA or employing the Cas9-sgRNA strategy for knockout (KO) in cultured endothelial cells resulted in impaired mitochondrial function and suppressed cell proliferation, migration, and in vitro capillary tube formation [20]. In vivo, endothelial knockdown of TIMM44 inhibited retinal angiogenesis [20], underscoring the importance of integral mitochondrial proteins in endothelial cell activation and angiogenesis.

RNA polymerase mitochondrial (POLRMT) is a crucial mitochondrial protein responsible for maintaining mitochondrial integrity and function [21–25]. POLRMT exerts a pivotal influence on oxidative phosphorylation (OXPHOS) and energy metabolism within mitochondria [26, 27]. It achieves this by orchestrating the transcription of genes encoded in mitochondrial DNA (mtDNA) that are crucial for the electron transport chain (ETC) and ATP synthase [28, 29], as well as by assisting in the initiation of mtDNA replication through the synthesis of RNA primers [28, 29]. This cooperative function with transcription factors including transcription factors A (TFAM) and B2 (TFB2M) ensure efficient mtDNA transcription and maintenance of mitochondrial genome integrity, ultimately guaranteeing the proficient operation of the ETC and the generation of ATP [30, 31]. Given the pivotal role of mitochondria in endothelial cell activation and angiogenesis, and recognizing POLRMT as a crucial mitochondrial protein, our study focused on the prospective involvement of POLRMT in angiogenesis, both in vitro and in vivo.

## Materials and methods

### Reagents, chemicals and antibodies

Polybrene, cell culture medium, serum, CCK-8 reagent, puromycin and other agents were provided by Sigma-Aldrich (St. Louis, MO). The antibodies and mRNA primers were from Dr. Shi at Soochow University [32]. All fluorescence dyes were purchased from Thermo-Fisher Invitrogen (Shanghai, China). The non-competitive POLRMT inhibitor IMT1 (inhibitor of mitochondrial transcription I) was from Dr. Li [21].

### Cells

HUVECs, hRMECs and hCMEC/D3 were reported previously [33–35] and were cultivated as described [33–36]. Endothelial cells were maintained under the pro-angiogenic state [33, 34]. Cells were subject to mycoplasma and microbial contamination examination. STR, population doubling time, morphology were examined to verify their genotypes.

### POLRMT silencing

Lentivirus-packed human POLRMT shRNAs (“sh-polrmt-s1” and “sh-polrmt-s2”, representing two different shRNA sequences) were from Dr. Shi [32] of Soochow University (Suzhou, China). The cultured endothelial cells (in polybrene-containing complete medium) were infected with the virus (at MOI = 15) for 48 h. Afterwards, cells were back to complete medium containing puromycin. Selection of stable endothelial cells lasted for 4–5 passages. The control endothelial cells were transduced with the lentivirus-packed scramble control non-sense shRNA (“shC”, also from Dr. Shi [32]). POLRMT expression, at both mRNA and protein levels, was always verified in the stable cells. For silencing POLRMT in vivo, the POLRMT shRNA (mouse, targeting: *AGGGTGAGCCCCTTATCCAGTTGGCCCATAACCTGGGCCTT*) sequence was sub-cloned into the AAV5-TIE1 construct (reported early [33, 34, 37]) to generate AAV.

### POLRMT knockout (KO)

The stable HUVECs with the Cas9-expressing construct (reported in our previous study [20]) were cultivated in polybrene-containing complete medium and were infected with lentivirus-packed CRISPR/Cas9-POLRMT-KO construct (from Dr. Wang [22]). The infected cells were back to complete medium containing puromycin. Selection of stable endothelial cells lasted for 4–5 passages. Next, stable cells were placed into 96-well plates and POLRMT KO was verified through PCR assays in each well. Lastly, the single stable POLRMT KO HUVECs were established and these cells were named as “ko-polrmt” HUVECs. In control HUVECs, cells were stably transduced with lentiviral CRISPR/Cas9-control construct (“koC”).

### POLRMT overexpression

HUVECs were seeded into six-well plates at 60% confluence in polybrene-containing complete medium and were infected with lentivirus encoding the POLRMT-overexpressing construct (also from Dr. Shi at Soochow University [32]) at MOI = 15. Afterwards, cells were back to complete medium containing puromycin. After 4–5 passages, two stable cell selections, “oe-polrmt-slc1” and “oe-polrmt-slc2”, were established. The control HUVECs were stably transduced with lentiviral empty vector (“LV”). POLRMT expression, at both mRNA and protein levels, was always checked in the stable cells.

### Functional assays and gene/protein detections

The nuclear EdU incorporation assay of cell proliferation, “Transwell” cell in vitro migration/invasion assays, capillary tube formation, DCF-DA/CellROX fluorescence staining of reactive oxygen species (ROS) production, JC-1 fluorescence staining of mitochondrial depolarization, the Caspase-3 activity assay, the nuclear TUNEL fluorescence staining of cell apoptosis and single strand DNA (ssDNA) as well as the mitochondrial complex I activity assay and cellular ATP detection were described in detail in our previous studies [20, 34, 35]. Thiobarbituric acid reactive substance (TBAR) assaying of lipid peroxidation and measuring superoxide dismutase (SOD) activity in fresh mouse retinal lysates were described in the previous study as well [20]. The protocols of histone DNA ELISA, quantitative real-time PCR (qRT-PCR) and Western blotting were described in other studies [34, 35, 38–41].

### Animal studies

The adult C57BL/6 mice were described early [20]. Approximately 0.12 µL AAV or IMT1 (or vehicle control) was intravitreously injected into the vitreous cavity as described [33, 34]. NeuN immunofluorescence staining of retinal ganglion cells (RGCs), hematoxylin-eosin (HE) staining, isolectin B4 (IB4) staining of retinal vasculature and retinal trypsin digestion assaying of acellular capillaries were described in previous studies [20, 33, 34]. βIII-tubulin (Tubb3)/NeuN fluorescence staining in the flat-mounted retinal gang cell layer (GCL) were described previously [42, 43]. Streptozotocin (STZ) injection and diabetic retinopathy (DR) mice were reported early [44]. C57BL/6 mice were fasted and intraperitoneally (*i.p.*) injected with STZ for five consecutive days. Mice with blood glucose level over 300 mg/dL were diabetic. “Mock” mice were injected with citrate buffer. Retinal vascular leakage assay by Evans blue (EB) staining was also described previously [34, 45]. The Institutional Animal Care and Use Committee and the Ethic Committee of Soochow University approved the protocols, and in according to the ARVO (Association for Research in Vision and Ophthalmology) statement.

### Human tissues

The tissues from written-informed consent patients were reported in the previous studies [34, 35]. Six patients with proliferative diabetic retinopathy (PDR) undergoing lensectomy combined with vitrectomy surgery and three age-matched patients undergoing surgery for traumatic retinectomy were enrolled. Each participant provided written informed consent. The anterior hyperplastic retinal membranes from the PDR patients were carefully removed, and fresh tissue specimens were obtained. The traumatic normal retinas were preserved as well. The protocols were approved by the Ethics Committee of Soochow University.

### Statistical analyses

Data, expressed as means ± standard deviation (SD), were all normally-distributed. One-way ANOVA plus a Scheffe’s f-test (for comparison of three or more groups, SPSS 23.0), or the two-tailed unpaired t test (for comparison of two groups, Excel 2007), were utilized to examine significance. ***P*** values < 0.05 were considered statistically significant.

## Results

### Genetic depletion of POLRMT induces anti-angiogenic activity in cultured endothelial cells

**Fig. 1:**
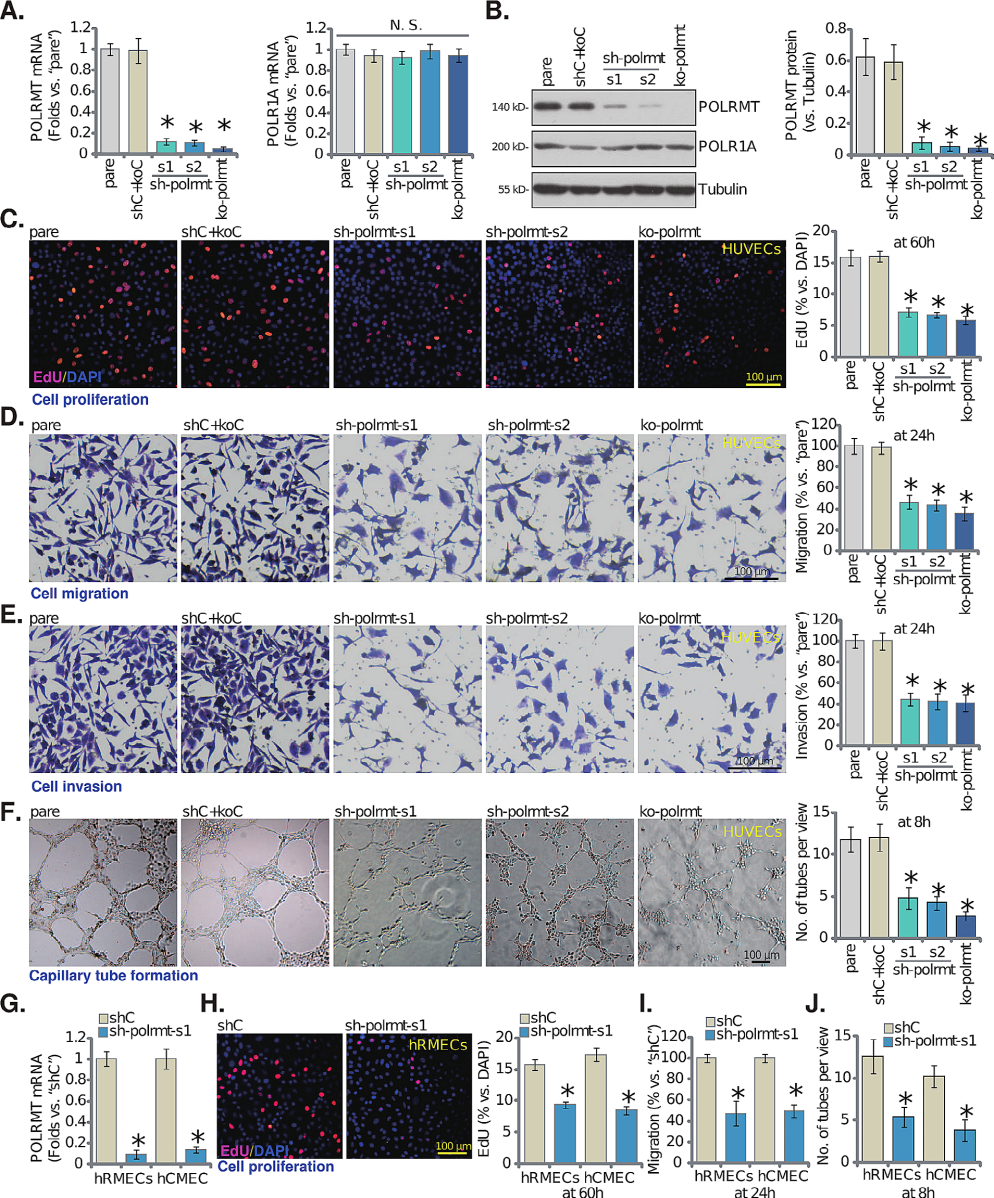
Genetic depletion of POLRMT induces anti-angiogenic activity in cultured endothelial cells. Expression of listed mRNAs and proteins in HUVECs with the described POLRMT shRNA (“sh-polrmt-s1” and “sh-polrmt-s2”), the CRISPR/Cas9-POLRMT-KO construct (“ko-polrmt”), or scramble non-sense shRNA plus CRISPR/Cas9-control construct (“shC + koC”) was shown (**A** and **B**); HUVECs (maintained under the pro-angiogenic state) were further cultured for designated times, cell proliferation (EdU incorporation in nuclei, **C**), migration (**D**), invasion (**E**) and capillary tube formation (**F**) were examined. *POLRMT* mRNA expression in hRMECs or hCMEC/D3 endothelial cells with sh-polrmt-s1 or control scramble non-sense shRNA (“shC”) was shown (**G**); After culture under the pro-angiogenic state for designated times, cell proliferation (EdU incorporation in nuclei, **H**), migration (**I**) and capillary tube formation (**J**) were examined. “Pare” stands for the parental control cells. Data were presented as mean ± standard deviation (SD, *n* = 5). * *P* < 0.05 versus “Pare”/“shC” cells. “N. S.” stands for non-statistical differences (*P* > 0.05, **A**). The experiments were repeated five times with similar results obtained. Scale bar = 100 μm

To explore the potential function of POLRMT in angiogenesis, genetic strategies were employed to deplete PLORMT in endothelial cells. Specifically, the lentivirus-packed POLRMT shRNAs, “sh-polrmt-s1” and “sh-polrmt-s2” (with non-overlapping different sequences [32]), were added separately to cultured HUVECs [20], and stable HUVECs established following puromycin selection. CRISPR/Cas9 KO knockout (KO) offers a precise method for completely inactivating a gene, helping validate the effects observed with shRNA by ruling out partial knockdowns and possible off-target effects. Thus, the Cas9-expressing stable HUVECs [20] were further infected with lentivirus-packed CRISPR/Cas9-POLRMT-KO construct [22], and stable cells again established following puromycin treatment and KO verification. These cells were named as “ko-polrmt” HUVECs. POLRMT expression was tested in the endothelial cells. As shown, expression of *POLRMT* mRNA (Fig. 1A) and protein (Fig. 1B) was substantially decreased in sh-polrmt-s1/2 HUVECs and ko-polrmt HUVECs. POLR1A functions in the nucleus as a subunit of RNA polymerase I, transcribing ribosomal RNA crucial for protein synthesis. We showed that the control POLR1A expression was unaltered by POLRMT shRNA/KO treatment (Fig. 1A and **B**). The control genetic treatments, scramble non-sense shRNA plus CRISPR/Cas9-control construct (“shC + koC”), failed to alter POLRMT and POLR1A expression in HUVECs (Fig. 1A and **B**).

Next, we explored the potential role of POLRMT depletion on the functions of HUVECs. As shown, in sh-polrmt-s1/2 HUVECs and ko-polrmt HUVECs, the EdU nuclear incorporation was substantially decreased (Fig. 1C), suggesting that POLRMT depletion impeded HUVEC proliferation (Fig. 1C). Moreover, “Transwell” assay results demonstrated that HUVEC in vitro migration (Fig. 1D) and invasion (Fig. 1E) were robustly slowed after POLRMT silencing or KO. In addition, the capillary tube formation of HUVECs was remarkably inhibited after POLRMT depletion (Fig. 1F). The control shC + koC treatment, as expected, did not alter proliferation, migration, invasion and capillary tube formation in HUVECs (Fig. 1C-F).

The lentivirus-packed sh-polrmt-s1 was also added to other endothelial cells, including human microvascular endothelial cells (hRMECs) and human cerebral microvascular endothelial cells (hCMEC/D3) [34], causing robust *POLRMT* mRNA downregulation (Fig. 1G). In hRMECs and hCMEC/D3, silencing of POLRMT similarly suppressed cell proliferation (EdU incorporation in nuclei, Fig. 1H), migration (Fig. 1I) and capillary tube formation (Fig. 1J). These results showed that POLRMT depletion induced anti-angiogenic activity in cultured endothelial cells.

### Genetic depletion of POLRMT impairs mitochondrial functions in cultured endothelial cells

**Fig. 2:**
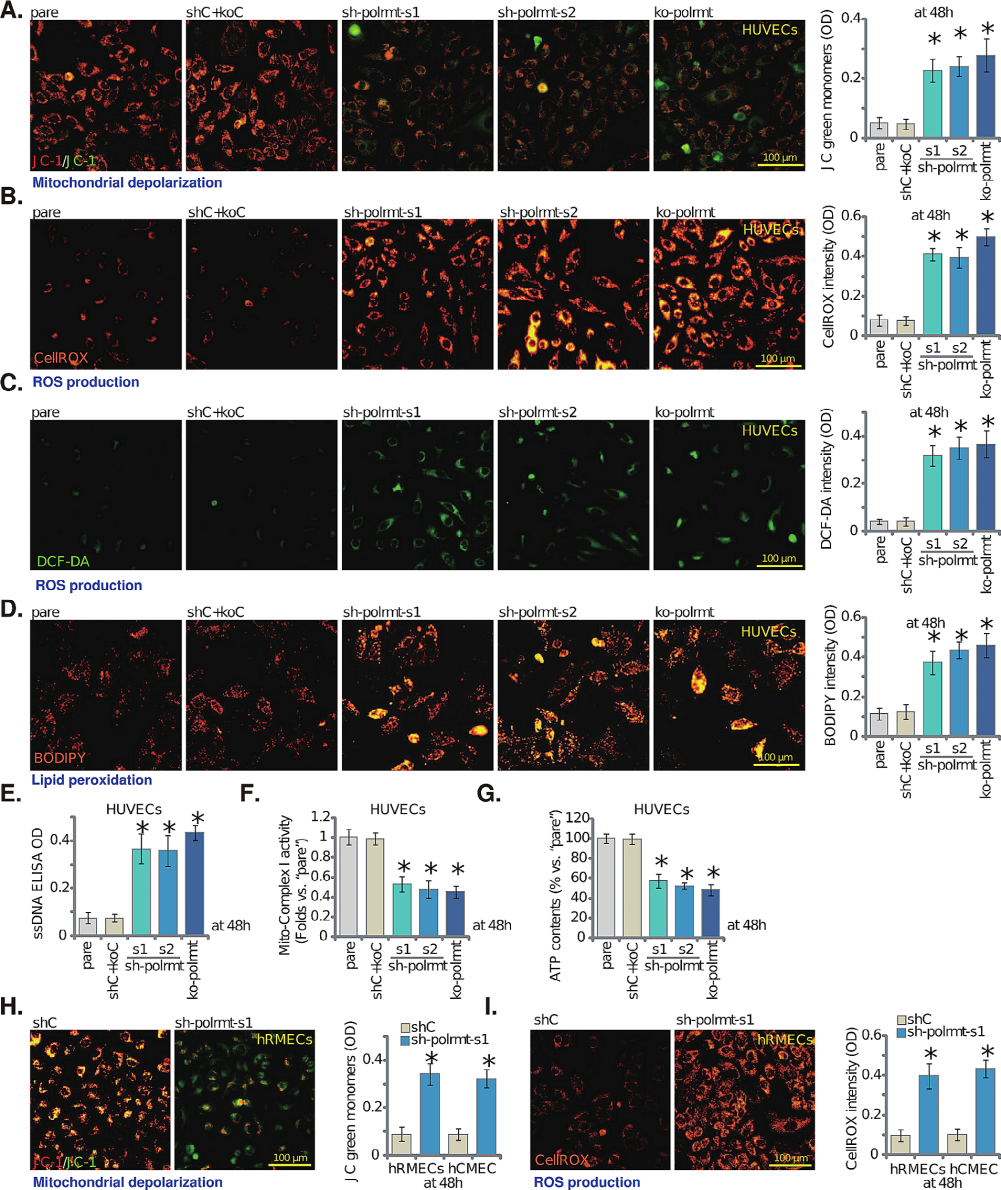
Genetic depletion of POLRMT impairs mitochondrial functions in cultured endothelial cells. HUVECs with the described POLRMT shRNA (“sh-polrmt-s1” and “sh-polrmt-s2”, representing two different shRNAs), the CRISPR/Cas9-POLRMT-KO construct (“ko-polrmt”), or scramble non-sense shRNA plus CRISPR/Cas9-control construct (“shC + koC”) were cultured for 48 h, the mitochondrial membrane potential reduction (mitochondrial JC-1 staining, **A**), ROS contents (CellROX and DCF-DA staining, **B** and **C**) and lipid peroxidation (BODIPY staining, **D**), ssDNA contents (**E**), the mitochondrial respiratory chain complex I activity (**F**) and cellular ATP contents (**G**) were examined. hRMECs or hCMEC/D3 endothelial cells, with sh-polrmt-s1 or control scramble non-sense shRNA (“shC”), were cultured for 48 h, mitochondrial depolarization (**H**) and ROS production (**I**) were examined similarly. “Pare” stands for the parental control cells. Data were presented as mean ± standard deviation (SD, *n* = 5). * *P* < 0.05 versus “Pare”/“shC” cells. The experiments were repeated five times with similar results obtained. Scale bar = 100 μm

Studies have shown that POLRMT is essential in maintaining mitochondrial functions and integrity, including mitochondrial DNA (mtDNA) transcription, OXPHOS, ATP synthesis and mitochondrial biogenesis [21, 22, 32, 46, 47]. We therefore explored whether genetic depletion of POLRMT disrupted mitochondrial functions in endothelial cells. In cultured HUVECs, POLRMT silencing (by “sh-polrmt-s1” and “sh-polrmt-s2”, see Fig. 1) or KO (“ko-polrmt”, see Fig. 1) induced mitochondrial depolarization (Fig. 2A), causing mitochondrial JC-1 green monomers accumulation (Fig. 2A). Moreover, mitochondrial oxidative stress was detected in POLRMT-depleted HUVECs, as POLRMT shRNA or KO led to substantial increases of CellROX red fluorescence intensity (Fig. 2B) and DCF-DA green fluorescence intensity (Fig. 2C). Increased lipid oxidation, evidenced by augmented BODIPY staining, was detected as well in POLRMT-silenced/-KO HUVECs (Fig. 2D). The accumulation of ssDNA supported increased DNA damage in HUVECs with POLRMT silencing/KO (Fig. 2E). Importantly, genetic depletion of POLRMT disrupted OXPHOS and energy production. As POLRMT shRNA or KO decreased mitochondrial respiratory chain complex I activity (Fig. 2F). Consequently, the ATP contents were decreased in POLRMT-depleted cells (Fig. 2G). The control shC + koC genetic treatment failed to significantly alter mitochondrial functions in HUVECs (Fig. 2A-G). In hRMECs and hCMEC/D3 endothelial cells, silencing of POLRMT by sh-polrmt-s1 (see Fig. 1) similarly induced mitochondrial depolarization (JC-1 green monomers accumulation, Fig. 2H) and ROS production (CellROX intensity increasing, Fig. 2I). Therefore, genetic depletion of POLRMT impaired mitochondrial functions in endothelial cells.

### Genetic depletion of POLRMT induces moderate but significant apoptosis in cultured endothelial cells

**Fig. 3:**
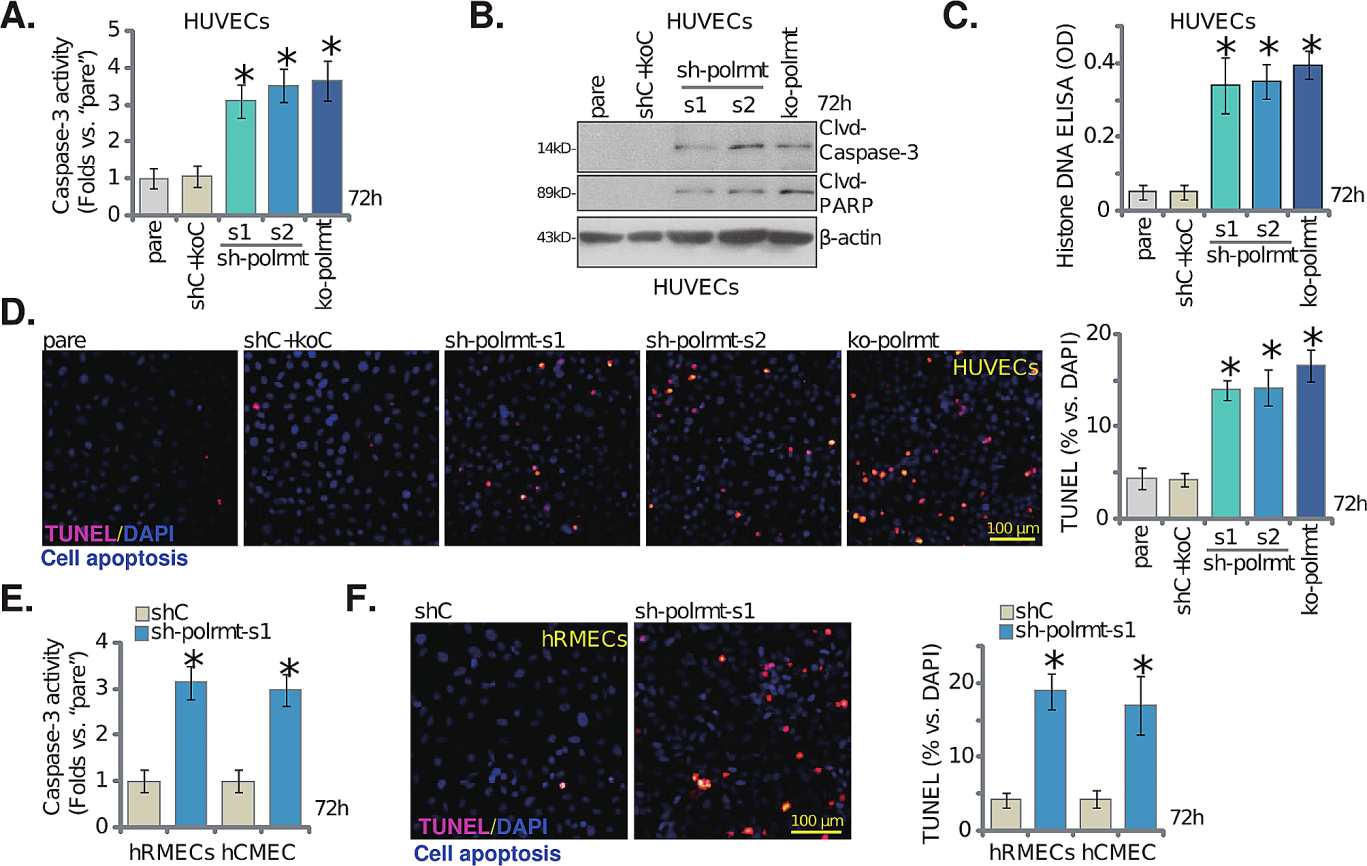
Genetic depletion of POLRMT induces moderate but significant apoptosis in cultured endothelial cells. HUVECs with the described POLRMT shRNA (“sh-polrmt-s1” and “sh-polrmt-s2”), the CRISPR/Cas9-POLRMT-KO construct (“ko-polrmt”), or scramble non-sense shRNA plus CRISPR/Cas9-control construct (“shC + koC”) were cultured for 72 h, the caspase-3 activity (**A**), expression of listed apoptosis proteins (**B**), histone-bound DNA (ELISA assays, **C**) and cell apoptosis (nuclear TUNEL assays, **D**) were examined. hRMECs or hCMEC/D3 endothelial cells with sh-polrmt-s1 or control scramble non-sense shRNA (“shC”) were cultured for indicated time, the caspase-3 activity (**E**) and apoptosis (nuclear TUNEL assay, **F**) were tested as well. “Pare” stands for the parental control cells. Data were presented as mean ± standard deviation (SD, *n* = 5). * *P* < 0.05 versus “Pare”/“shC” cells. The experiments were repeated five times with similar results obtained. Scale bar = 100 μm

Since POLRMT depletion (shRNA/KO) disrupted mitochondrial functions and led to energy stress in endothelial cells, we explored its role on cell apoptosis. As shown, POLRMT silencing (by “sh-polrmt-s1” and “sh-polrmt-s2”) or KO (“ko-polrmt”) enhanced the caspase-3 activity in HUVECs (Fig. 3A). Moreover, POLRMT shRNA/KO in HUVECs increased the cleavages of caspase-3, caspase-9 and poly (ADP-ribose) polymerase 1 (PARP) (Fig. 3B). The Histone-bound DNA contents, an indicator of apoptosis induction, was increased in POLRMT-depleted HUVECs (Fig. 3C). Supporting apoptosis activation, we found that POLRMT shRNA or KO increased TUNEL nuclei number (Fig. 3D). Interestingly, POLRMT depletion only induced moderate cell apoptosis and less than 20% cells were apoptotic after POLRMT depletion (Fig. 3D). The control shC + koC treatment failed to induce caspase-apoptosis activation in HUVECs (Fig. 3A-D). In hRMECs and hCMEC/D3 endothelial cells, sh-polrmt-s1-induced silencing of POLRMT (see Figs. 1 and 2) increased the caspase-3 activity (Fig. 3E) and TUNEL nuclei number (Fig. 3F) as well. These results supported that POLRMT depletion induced apoptosis activation in endothelial cells.

### The POLRMT inhibitor IMT1 induces anti-angiogenic activity in cultured endothelial cells

**Fig. 4:**
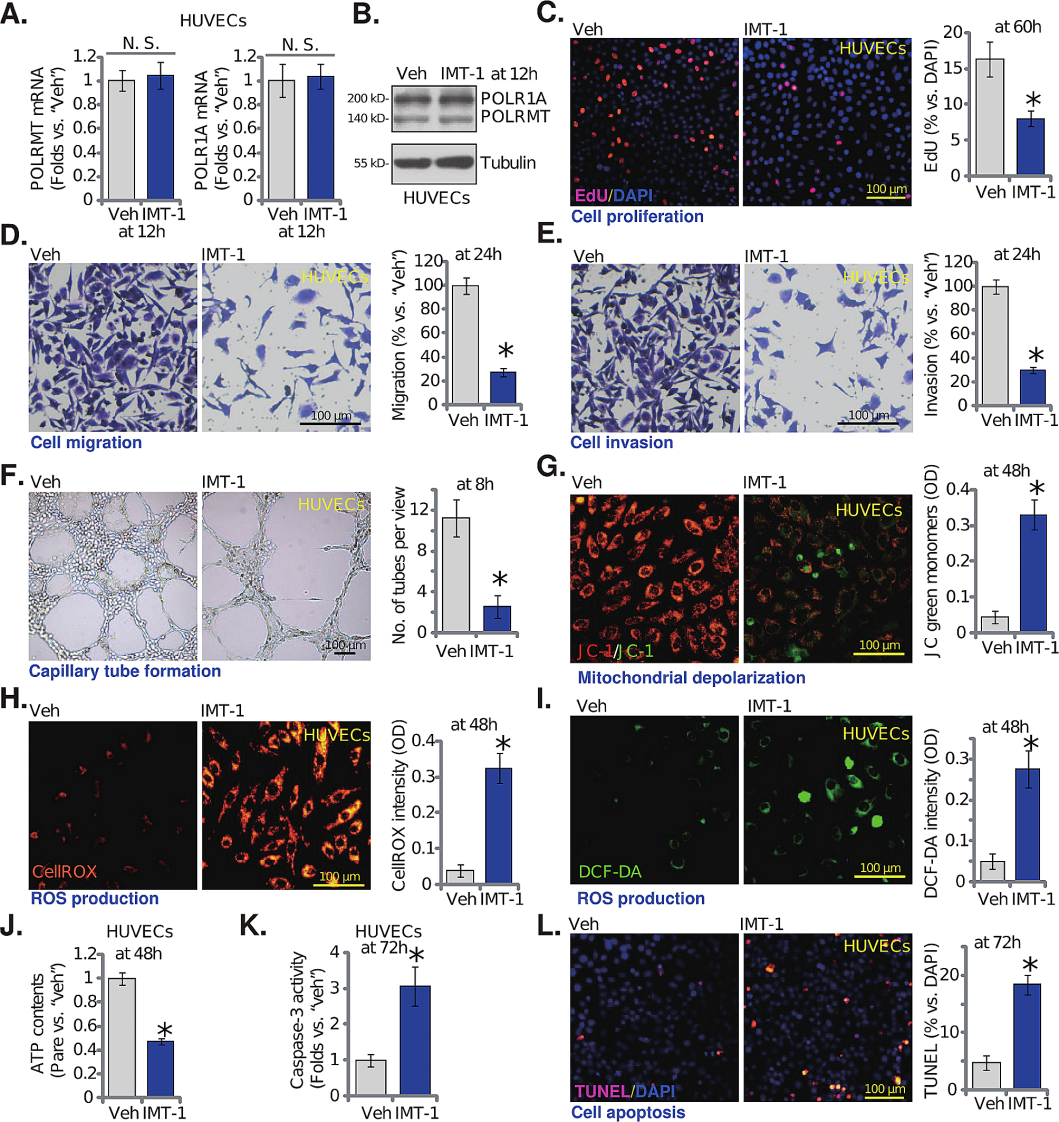
The POLRMT inhibitor IMT1 induces anti-angiogenic activity in cultured endothelial cells. Culture HUVECs were treated with the POLRMT inhibitor IMT1 (0.5 µM) or the vehicle control (0.1% DMSO, “Veh”), cells were further cultured for indicated time periods, expression of listed mRNAs and proteins was shown (**A** and **B**); Cell proliferation (EdU incorporation, **C**), migration (**D**), invasion (**E**) and capillary tube formation (**F**) were examined; The mitochondrial membrane potential reduction (JC-1 staining, **G**), ROS contents (CellROX and DCF-DA fluorescence staining, **H** and **I**) and ATP contents (**J**) were examined as well. The caspase-3 activity (**K**) and nuclear TUNEL staining (**L**) were also tested. Data were presented as mean ± standard deviation (SD, *n* = 5). * *P* < 0.05 versus “Veh” cells. The experiments were repeated five times with similar results obtained. Scale bar = 100 μm

We next explored the potential effect of IMT1, a first-in-class POLRMT inhibitor [21, 22], on endothelial cells. In cultured HUVECs, treatment with IMT1 at 0.5 µM (concentration was based on previous studies [21, 22], for 12 h) failed to alter POLRMT and POLR1A expression (Fig. 4A and **B**). The POLRMT inhibitor however hindered HUVEC proliferation by inhibiting nuclear EdU incorporation (Fig. 4C). IMT1, at the applied concentration, also potently impeded HUVEC in vitro migration (Fig. 4D) and invasion (Fig. 4E). In addition, the capillary tube formation of HUVECs was significantly suppressed following treatment with IMT1 (Fig. 4F).

The POLRMT inhibitor impaired mitochondrial functions in HUVECs and induced mitochondrial depolarization (accumulation of green JC-1 monomers, Fig. 4G). ROS production was augmented in IMT1-treated HUVECs, as the red CellROX fluorescence intensity (Fig. 4H) and the green DCF-DA fluorescence intensity (Fig. 4I) were both strengthened. Contrarily, the cellular ATP contents were downregulated following treatment with the POLRMT inhibitor in HUVECs (Fig. 4J). Moderate but significant apoptosis activation was also detected in IMT1-stimulated HUVECs, where the caspase-3 activity (Fig. 4K) and the TUNEL nuclei number (Fig. 4L) were both increased. Therefore, IMT1 impaired mitochondrial functions and induced anti-angiogenic activity, further supporting the role of POLRMT in angiogenesis in vitro.

### Forced POLRMT overexpression induces pro-angiogenic activity in cultured endothelial cells

**Fig. 5:**
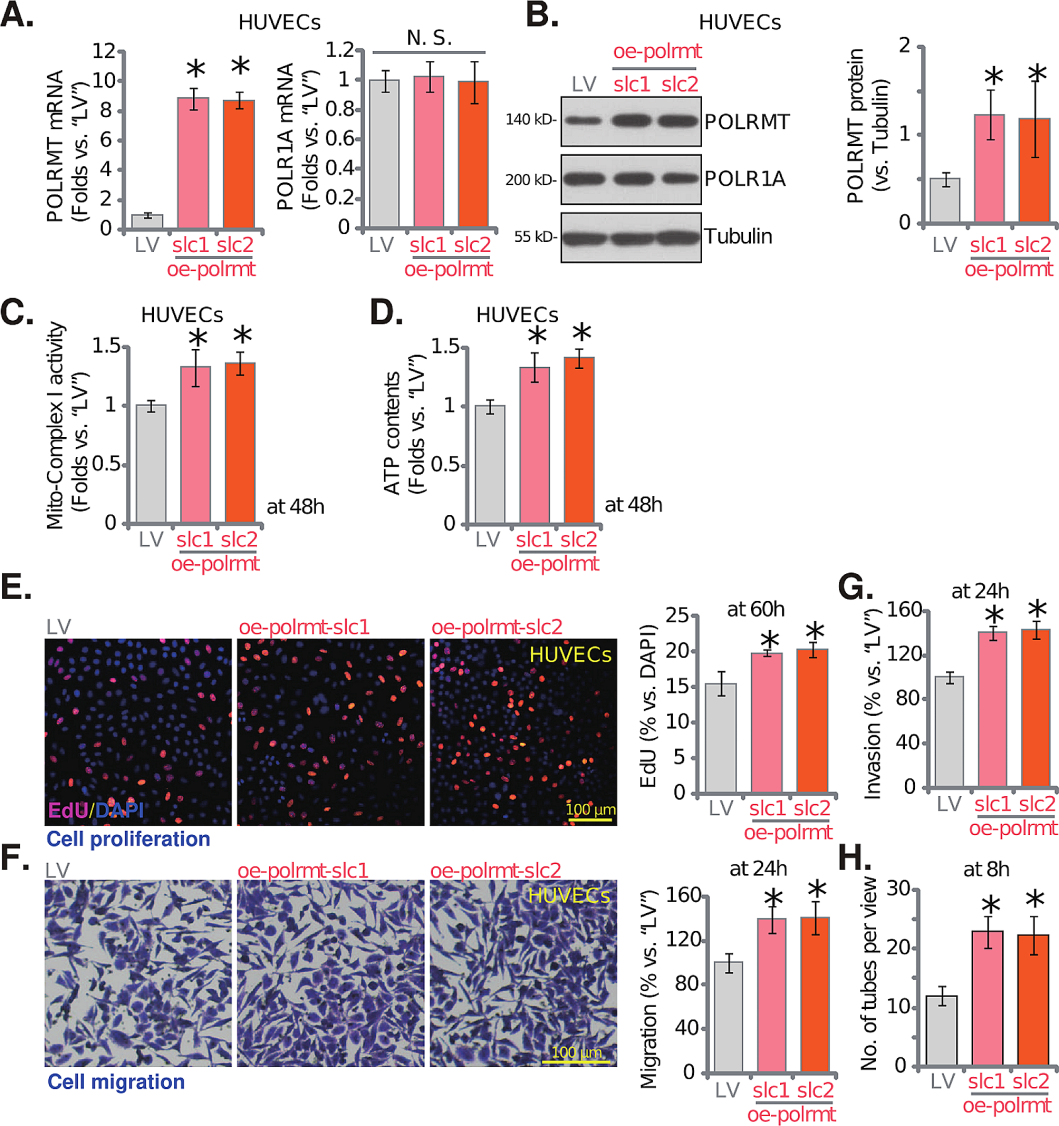
Forced POLRMT overexpression induces pro-angiogenic activity in cultured endothelial cells. Expression of listed mRNAs and proteins in HUVECs with lentivirus-packed POLRMT-overexpressing construct (“oe-polrmt-slc1” and “oe-polrmt-slc2”, representing two different stable selections) or the lentiviral vector (“LV”) was shown (**A** and **B**); Cells were further cultured for indicated time periods, the mitochondrial respiratory chain complex I activity (**C**) and cellular ATP contents (**D**) were examined; Cell proliferation (EdU incorporation, **E**), migration (**F**), invasion (**G**) and capillary tube formation (**H**) were examined as well. * *P* < 0.05 versus “LV” cells. “N. S.” stands for non-statistical differences (*P* > 0.05, **A**). The experiments were repeated five times with similar results obtained. Scale bar = 100 μm

Since POLRMT depletion or inhibition led to anti-angiogenic activity in cultured endothelial cells, we thus hypothesized that overexpression of POLRMT should exert pro-angiogenic actions. Therefore the lentivirus-packed POLRMT-overexpressing construct (from Dr. Shi [32]) was added to HUVECs. HUVECs were then treated with puromycin and two stable cell selections, “oe-polrmt-slc1” and “oe-polrmt-slc2”, were established. As compared to control HUVECs with lentiviral empty vector (“LV”), mRNA and protein expression of POLRMT was significantly elevated in oe-polrmt-slc1/2 HUVECs (Fig. 5A and **B**), where POLR1A expression was unaltered (Fig. 5A and **B**). The mitochondrial respiratory chain complex I (Fig. 5C) and ATP contents (Fig. 5D) were strengthened in POLRMT-overexpressed HUVECs. POLRMT overexpression potentiated HUVEC proliferation and augmented nuclear EdU incorporation (Fig. 5E). The in vitro cell migration (Fig. 5F) and invasion (Fig. 5G) were accelerated as well after forced POLRMT overexpression in HUVECs. The capillary tube formation ability was augmented in POLRMT-overexpressed HUVECs (Fig. 5H). These results supported that POLRMT overexpression induced pro-angiogenic activity in HUVECs.

### Endothelial knockdown of POLRMT inhibits retinal angiogenesis in vivo

**Fig. 6:**
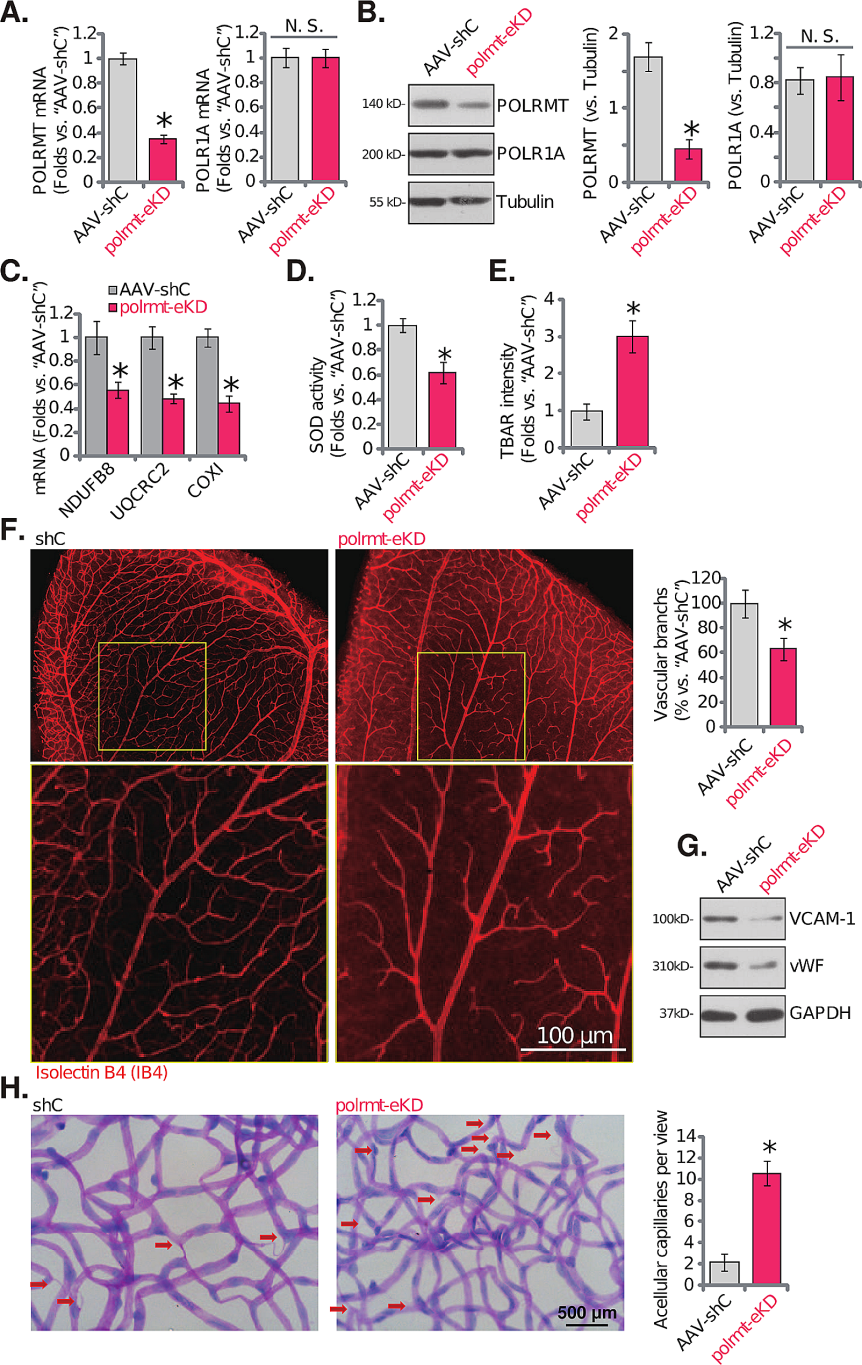
Endothelial knockdown of POLRMT inhibits retinal angiogenesis in vivo. The adult C57BL/6 mice were intravitreously injected with murine AAV5-TIE1-POLRMT shRNA (“polrmt-eKD”, 0.12 µL) or AAV5-packed scramble control shRNA (“AAV-shC”, 0.12 µL). Fifteen days later, the murine retinal tissues were collected and listed mRNAs and proteins in the fresh tissue lysates were tested (**A**-**C** and **G**); The SOD activity (**D**) and the TBAR intensity (**E**) were examined as well. Alternatively, the retinal slides were obtained and the retinal vasculatures were visualized via retinal isolectin B4 (IB4) staining (**F**); The acellular capillaries (red arrows) were visualized via the retinal trypsin digestion assays (**H**). The data were presented as mean ± standard deviation (SD, *n* = 5). * *P* < 0.05 vs. “AAV-shC” group. “N. S.” stands for non-statistical differences (*P* > 0.05, **A** and **B**). The experiments were repeated five times with similar results obtained. Scale bar = 100 μm (**F**). Scale bar = 500 μm (**H**)

In order to explore the potential role of POLRMT on angiogenesis in vivo, the mouse retinal vasculature experiments were carried out (as described in our previous study [20]). The adult mice were first intravitreously injected with murine AAV5-TIE1-POLRMT shRNA. The latter contained the sequence of the endothelial cell specific promoter TIE1 [33, 34]. It led to endothelial knockdown of POLRMT (“polrmt-eKD”). The murine AAV5-TIE1-scramble control shRNA (“AAV-shC”) was injected as the genetic control treatment (see previously [20]). The murine retinal tissues were collected 15 days after virus injection and tissue lysates were tested. Expression of *POLRMT* mRNA (Fig. 6A) and protein (Fig. 6B) was decreased in the retinal tissues of polrmt-eKD mice, where *POLR1A* mRNA (Fig. 6A) and protein (Fig. 6B) expression was unaltered. POLRMT-dependent mitochondrial transcripts, *NDUFB8*, *UQCRC2* and *COXI* [22, 26, 32, 48, 49], were decreased in retinal tissues of polrmt-eKD mice (Fig. 6C). With endothelial knockdown of POLRMT, the SOD activity was decreased (Fig. 6D) in retinal tissues and TBAR intensity (Fig. 6E) was increased, supporting mitochondrial injury and oxidative stress.

The IB4 staining of retinal vasculature showed that endothelial knockdown of POLRMT robustly inhibited angiogenesis in mouse retina, as polrmt-eKD mice displayed significantly reduced number of retinal vascular branches and branch points (Fig. 6F). Two endothelial marker proteins, von willebrand factor (vWF) and VCAM-1 [20], were also downregulated in retinal tissues following polrmt-eKD (Fig. 6G). Testing acellular capillary formation, using the retinal trypsin digestion assays (Fig. 6H), further showed that endothelial knockdown of POLRMT inhibited angiogenesis and caused pathological angiogenesis.

### Intravitreous injection of IMT1 inhibits retinal angiogenesis in vivo

**Fig. 7:**
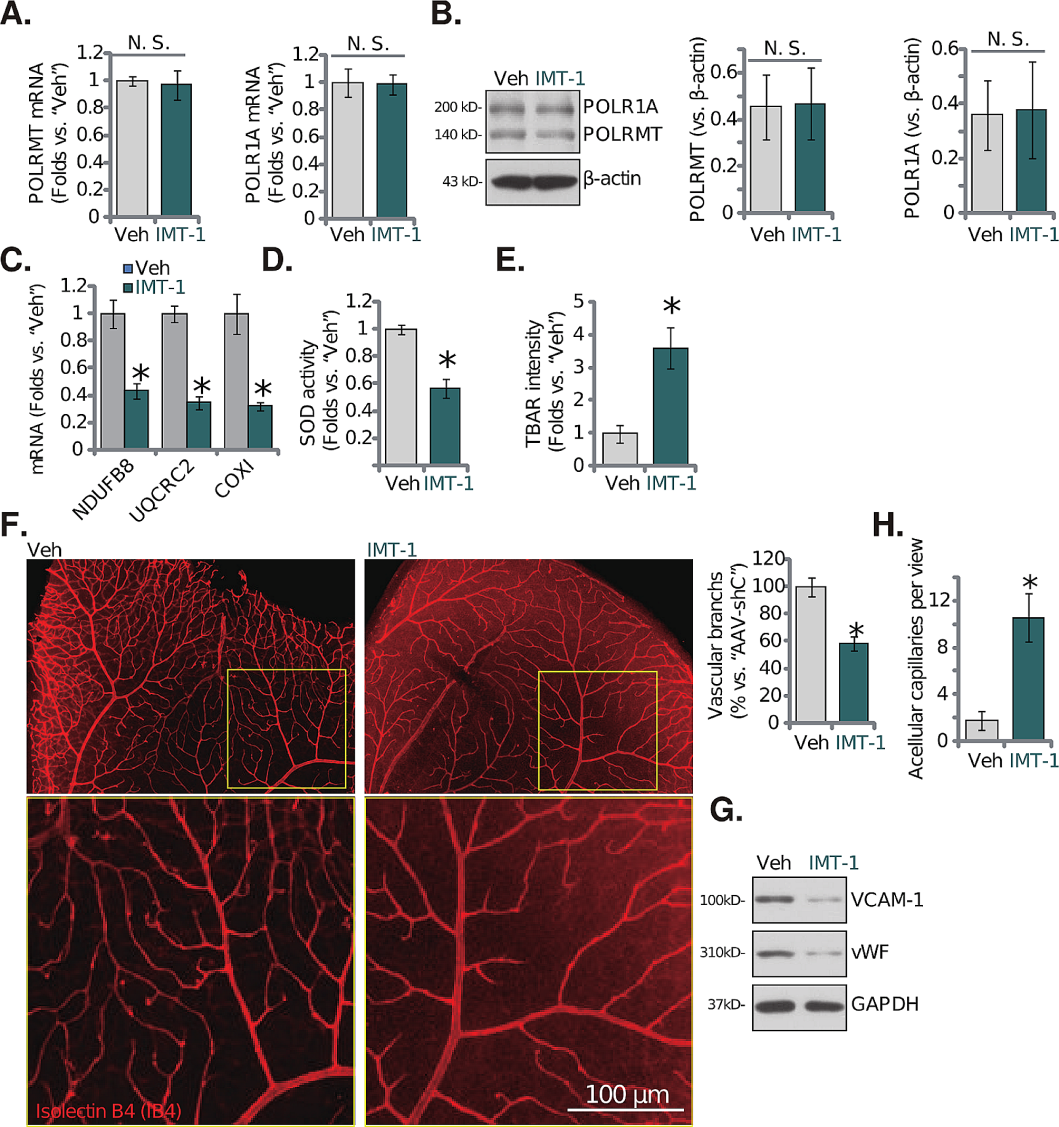
Intravitreous injection of IMT1 inhibits retinal angiogenesis in vivo. The adult C57BL/6 mice were intravitreously injected with IMT1 (0.25 nM, 0.12 µL) or the vehicle control (“Veh”, 0.12 µL) for 48 h, listed mRNAs and proteins in the fresh tissue lysates were tested (**A**-**C** and **G**); The SOD activity (**D**) and TBAR intensity (**E**) were examined as well. Alternatively, the retinal tissue slides were obtained and the retinal vasculatures were visualized via retinal isolectin B4 (IB4) staining (**F**); The acellular capillaries (red arrows) were visualized via the retinal trypsin digestion assays (**H**). The data were presented as mean ± standard deviation (SD, *n* = 5). “N. S.” stands for non-statistical differences (*P* > 0.05, **A** and **B**).* *P* < 0.05 vs. “Veh” group. The experiments were repeated five times with similar results obtained. Scale bar = 100 μm (**F**)

Thus, endothelial knockdown of POLRMT inhibited retinal angiogenesis in vivo, we next explored whether IMT1, the POLRMT inhibitor, could exert similar actions. The adult C57BL/6 mice were intravitreously injected with IMT-1 (0.25 nM) for 48 h and the retinal tissues were collected. As shown, mRNA and protein expression of POLRMT and POLR1A was not significantly altered after IMT-1 treatment (Fig. 7A and **B**). Contrarily, POLRMT-dependent mitochondrial transcripts, *NDUFB8*, *UQCRC2* and *COXI* [21, 22, 26, 32, 48, 49], were downregulated (Fig. 7C). The decreased SOD activity (Fig. 7D) and the increased TBAR intensity (Fig. 7E) supported mitochondrial dysfunction and oxidative injury in IMT1-treated mouse retinal tissues. IB4 staining of retinal vasculature showed that intravitreous injection of the POLRMT inhibitor disrupted retinal angiogenesis in vivo, with the number of vascular branches and branch points decreased (Fig. 7F). Expression of endothelial marker proteins, vWF and VCAM-1, was downregulated as well after IMT1 treatment in retinal tissues (Fig. 7G). Increased number of acellular capillaries further supported disruption of angiogenesis following POLRMT inhibition in murine retinas (Fig. 7H). Therefore, intravitreous injection of IMT1 inhibited retinal angiogenesis in vivo.

### POLRMT upregulation participates in pathological retinal angiogenesis in diabetic retinopathy mice

**Fig. 8:**
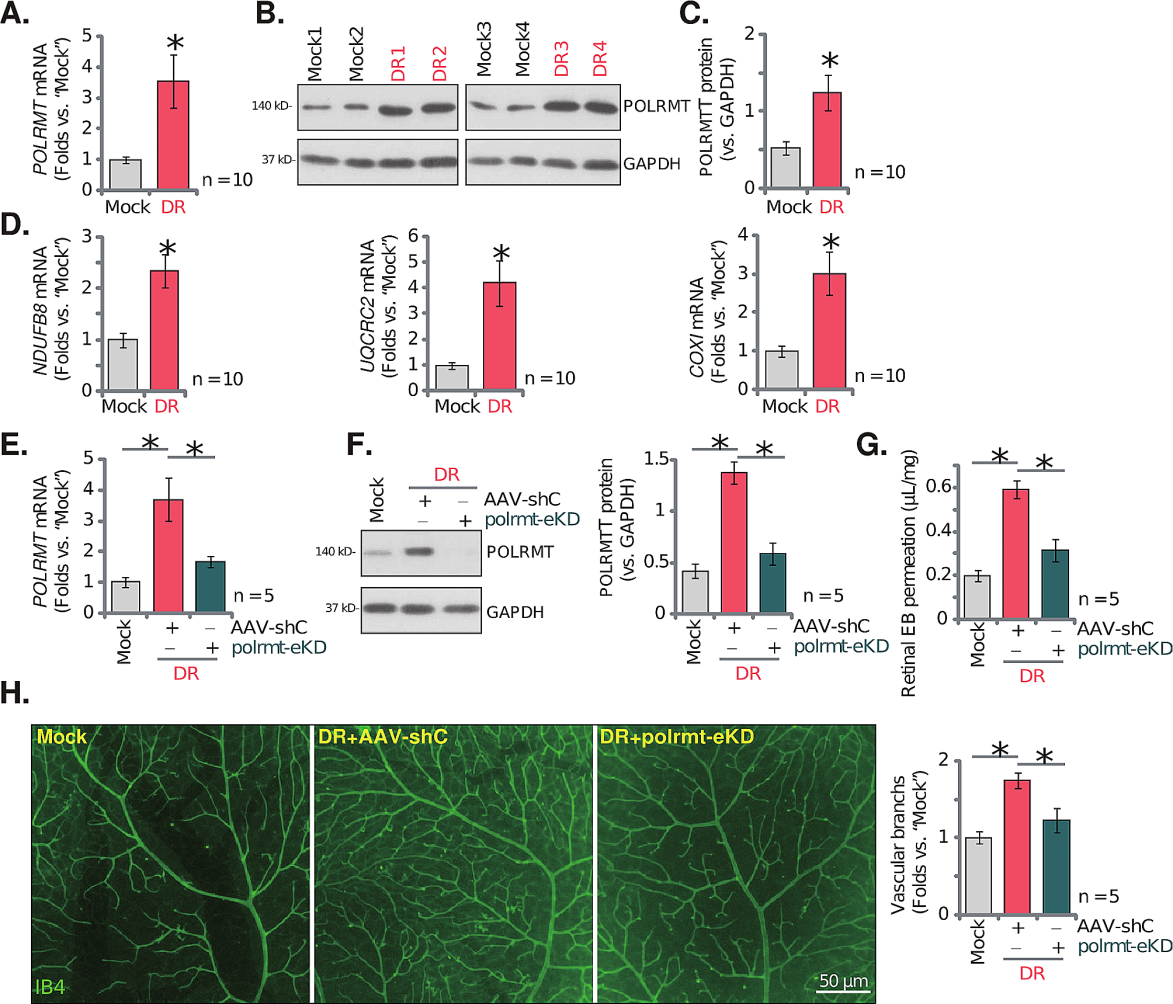
POLRMT upregulation participates in pathological retinal angiogenesis in diabetic retinopathy mice. Ninety (90) days after the last STZ administration, the retinal tissues of diabetic retinopathy (“DR”) mice and “Mock” control mice were collected, expression of listed mRNA and proteins was tested (**A**-**D**). Day-30 after the last STZ administration, mice were intravitreously injected with murine AAV5-TIE1-POLRMT shRNA (“polrmt-eKD”, 0.12 µL) or AAV5-packed scramble control shRNA (“AAV-shC”, 0.12 µL); After another 60 days, POLRMT expression in the retinal tissues was examined (**E** and **F**). Alternatively, mice were infused with Evans blue (EB) for 2 h, with EB leakage quantified (**G**); IB4 staining was performed to visualize the retinal vasculature (**H**, scale bar = 50 μm). “Mock” stands for mice with citrate buffer administration. **A**-**D**, *n* = 10 mice per group. **E**-**H**, *n* = 5 mice per group. * *P* < 0.05 vs. “Mock” (**A**-**D**). * *P* < 0.05 (**E**-**H**)

Whether expression of POLRMT was changed in streptozotocin (STZ)-injected DR mice was tested. After 90 days of the last STZ administration, the retinal tissues of both DR mice and control mice were collected. In the retinal tissues of DR mice, *POLRMT* mRNA levels were significantly elevated (Fig. 8A). Moreover, upregulation of POLRMT protein in retinal tissues was detected in four STZ-administrated DR mice (Fig. 8B). After combining all 10 sets of the blotting data, we found that POLRMT protein upregulation was significant (Fig. 8C). Expression of POLRMT-dependent genes, including *NDUFB8*, *UQCRC2* and *COXI*, was upregulated as well in retinal tissues of DR mice (Fig. 8D).

To explore whether POLRMT upregulation was involved in the pathological retinal angiogenesis in DR mice, on day-30 after the last STZ administration, AAV5-TIE1-POLRMT shRNA was again intravitreously injected into retinas of DR mice (“polrmt-eKD”). The control DR mice were subjected to intravitreous injection of AAV-shC. After another 60 days, the fresh retinal tissues were collected and examined. As shown, *POLRMT* mRNA and protein expression was decreased in retinal tissues of polrmt-eKD DR mice (Fig. 8E and **F**).

**Fig. 9:**
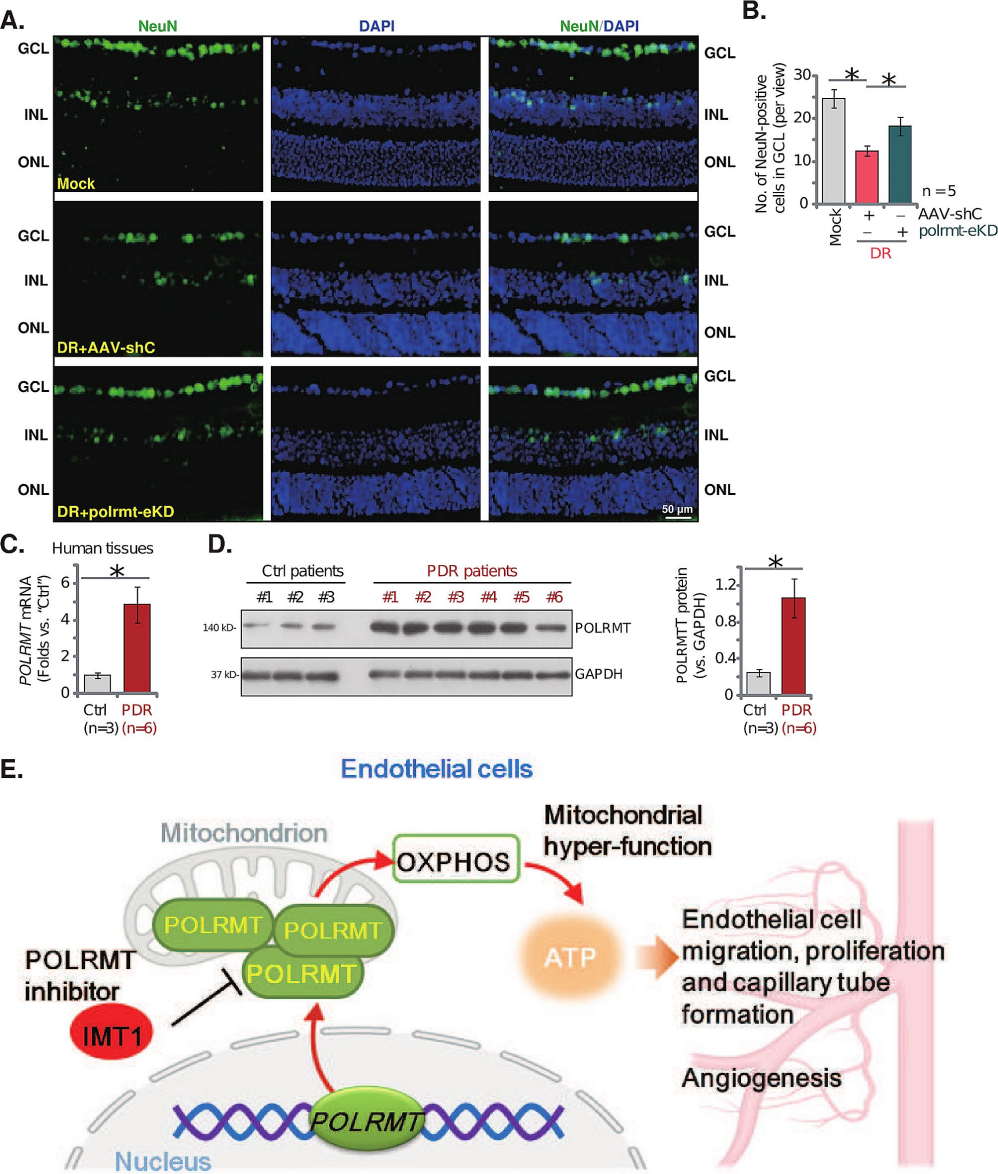
POLRMT expression is increased in proliferative membrane tissues of proliferative diabetic retinopathy (PDR) patients. Day-30 after the last STZ administration, DR mice were injected intravitreously with murine AAV5-TIE1-POLRMT shRNA (“polrmt-eKD”, 0.12 µL) or AAV5-packed scramble control shRNA (“AAV-shC”, 0.12 µL); After another 60 days, NeuN/DAPI immunofluorescence staining in the retinal slides was shown (**A**, scale bar = 50 μm) and NeuN-positive RGCs in GCL were quantified (**B**, *n* = 5 mice per group). The listed human tissues were homogenized and *POLRMT* mRNA and protein expression was examined (**C** and **D**, *n* = 3/6). The proposed signaling carton of this study (**E**).* *P* < 0.05

When compared to the Mock control mice, the retinal vascular leakage, quantified through Evans blue (EB) staining, was increased in AAV-shC DR mice (Fig. 8G). Moreover, IB4 staining results showed increased vascular complexity (angiogenesis) in retinas of DR mice (Fig. 8H). Importantly, polrmt-eKD largely inhibited pathological retinal angiogenesis in DR mice (Fig. 8G and **H**), inhibiting vascular leakage (Fig. 8G) and reducing vascular complexity (Fig. 8H). Furthermore, in AAV-shC DR mice the number of NeuN-stained RGCs in GCL was substantially decreased, supporting RGC degeneration (Fig. 9A and **B**). Notably, polrmt-eKD mitigated RGCs degeneration in DR mice (Fig. 9A and **B**). These results together supported that POLRMT upregulation participated in pathological retinal angiogenesis in DR mice.

### POLRMT expression is increased in proliferative membrane tissues of proliferative diabetic retinopathy (PDR) patients

Lastly, POLRMT expression in patients’ proliferative retinal tissues was tested. We tested the previously-described human tissues [34, 35, 44]. Retinal proliferative membrane tissues of six different human PDR patients (“PDR”) along with the retinal tissues of three age-matched traumatic retinectomy control patients (“Ctrl”) were obtained [34, 35]. As shown, *POLRMT* mRNA (Fig. 9C) and protein (Fig. 9D) expression was substantially increased human PDR patients’ proliferative retinal proliferative membrane tissues. These results further supported a possible role of POLRMT in pathological retinal angiogenesis of DR.

## Discussion

The integrity of mitochondria is crucial for endothelial cell activation and angiogenesis [17–19, 50, 51]. Wang et al., reported that endothelial knockdown of the mitochondrial outer-membrane protein FUNDC1 (FUN14 domain-containing protein 1) decreased VEGFR2 expression and inhibited tube formation, spheroid-sprouting in vitro and angiogenesis in vivo [17]. In endothelial progenitor cells, C3k-mediated blockage of pyruvate kinase muscle isoenzyme 2 (PKM2) downregulated expression of angiogenesis-associated genes and hindered tube formation [18]. Mitochondrial dysfunction and oxidative stress were observed in C3k-stimulated endothelial progenitor cells [18]. Our recent study has shown that genetic depletion or pharmacological blockage (by MB-10) of TIMM4, an inner mitochondrial membrane protein, led to mitochondrial protein input arrest and impeded angiogenesis in vitro and in vivo [20].

Recent studies have consistently affirmed the central role of POLRMT in mtDNA transcription, OXPHOS, energy production, and the proliferation of various cancer cell types [21–23, 26, 32, 48]. Han and colleagues demonstrated that elevated POLRMT levels were essential for the in vitro growth of osteosarcoma cells and the development of osteosarcoma xenografts in nude mice [48]. The depletion of POLRMT using genetic means resulted in mitochondrial dysfunction, energy depletion, and apoptosis in osteosarcoma cells [48]. In another study, Zhou and co-workers revealed that genetic depletion of POLRMT inhibited mitochondrial transcription, impaired mitochondrial function, and hindered the growth of non-small cell lung cancer cells (NSCLC) both in vitro and in vivo [32]. Wang et al. also documented the significance of POLRMT overexpression in skin squamous cell carcinoma (SCC) for sustaining mitochondrial hyperfunction and cell proliferation [22].

The findings of this study provide strong support for the importance of the mitochondrial protein POLRMT in endothelial cell activation and angiogenesis. Within cultured endothelial cells, including HUVECs, hRMECs, and hCMEC/D3, the introduction of POLRMT shRNA or KO demonstrated robust anti-angiogenic effects, hampering cell proliferation, migration, and the formation of capillary tubes. Furthermore, there was a noteworthy induction of apoptosis in POLRMT-depleted endothelial cells. In contrast, the overexpression of POLRMT had a pro-angiogenic impact, enhancing endothelial cell proliferation, migration, and capillary tube formation. In vivo, knockdown of POLRMT in endothelial cells inhibited retinal angiogenesis. Therefore, these results underscored the significant role of POLRMT in angiogenesis.

A key discovery from this study emphasizes the critical role of POLRMT in preserving the integrity of mitochondria within endothelial cells. Silencing or KO of POLRMT had detrimental effects on mitochondrial function, leading to mitochondrial depolarization, increased production of ROS, oxidative stress, lipid oxidation, DNA damage, and a reduction in ATP levels. Conversely, the overexpression of POLRMT enhanced the activity of mitochondrial respiratory chain complex I and increased ATP levels in HUVECs. Additionally, endothelial knockdown of POLRMT induced oxidative stress and lipid oxidation in retinal tissues. These findings suggest that the promotion of angiogenesis by POLRMT could be attributed to its role in maintaining optimal mitochondrial functions in endothelial cells (Fig. 9E).

In recent studies, the development of IMT1, a first-in-class non-competitive inhibitor of POLRMT, has garnered attention [26, 52]. IMT1’s ability to inhibit POLRMT has been demonstrated to halt mitochondrial transcription and disrupt OXPHOS processes [21, 22, 52]. This POLRMT inhibitor has been shown to compromise mitochondrial functions and impede the growth of various cancer cell types, including endometrial carcinoma cells and skin squamous cell carcinoma cells [21, 22]. In our current investigation, we unveiled that IMT1-induced inhibition of POLRMT elicited anti-angiogenic effects in vitro, effectively suppressing endothelial cell proliferation, migration, and capillary tube formation. Moreover, the intravitreous administration of IMT1 was found to induce mitochondrial dysfunction, oxidative damage in mouse retinal tissues and hinder retinal angiogenesis in vivo (see Fig. 9E). These outcomes further underscore the indispensable role of POLRMT in the regulation of angiogenesis.

DR is a multifaceted disorder characterized by two primary stages, non-proliferative diabetic retinopathy (NPDR) and proliferative diabetic retinopathy (PDR) [53–56]. The transition from NPDR to PDR is marked by the development of abnormal, fragile retinal blood vessels, which are prone to leakage, leading to retinal edema, hemorrhages, fibrovascular proliferation, and retinal detachment. This neovascularization is primarily elicited by chronic hyperglycemia-induced vascular damage, augmented by a milieu of inflammatory and oxidative stress factors [53–58]. The intricate interplay of these pathogenic elements triggers the upregulation of key angiogenic mediators, most notably VEGF, causing the formation of pathological blood vessels in the hypoxic retinal microenvironment [54, 59–61]. Therapeutic strategies for DR center on the amelioration of pathological angiogenesis and to understand the underlying mechanisms.

In the current study, we observed a significant upregulation of *POLRMT* mRNA and protein expression in the proliferative retinal membrane tissues obtained from PDR patients. This was further corroborated by the elevated levels of both *POLRMT* mRNA and protein expression, along with an increase in the expression of POLRMT-dependent genes, in the retinal tissues of murine models with STZ-induced diabetic retinopathy (DR). Of notable significance, we found that targeted silencing of POLRMT in retinal endothelial cells, achieved through intravitreous administration of AAV5-TIE1-POLRMT shRNA, effectively attenuated the pathological retinal angiogenesis and ameliorated the degeneration of RGCs in the DR murine models. In light of these findings, we propose that the augmented expression of POLRMT plays a pivotal role in pathological angiogenesis in DR, and it represents a promising and pertinent therapeutic target. (Fig. 9E).

### Electronic supplementary material

Below is the link to the electronic supplementary material.

Supplementary Material 1

## Data Availability

No datasets were generated or analysed during the current study.
