# Supplementary figures and images for: Identification of the central role of RNA polymerase mitochondrial for angiogenesis
Source: Cell Commun Signal. 2024 Jun 21;22:343. doi: 10.1186/s12964-024-01712-9 (PMC11191269; doi:10.1186/s12964-024-01712-9)

Figure S1: The uncropped images of the study.

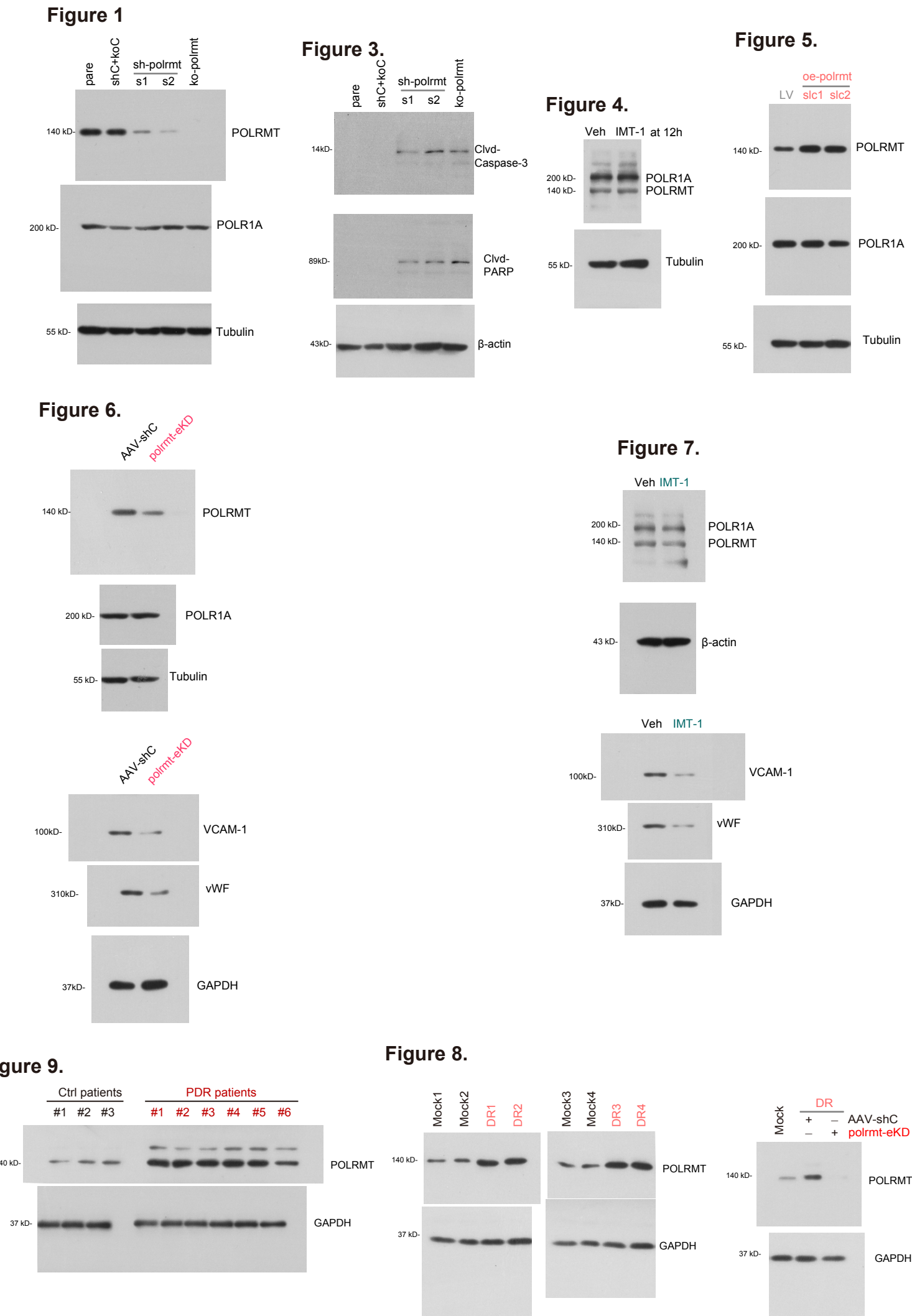

Supplement: Supplementary file 1 — Supplementary Material 1 [file 12964_2024_1712_MOESM1_ESM.pdf]
